# Decoding Genomic Diversity to Guide Tumor Lesion‐Specific Treatment of Multifocal Hepatocellular Carcinoma

**DOI:** 10.1002/cam4.70814

**Published:** 2025-03-27

**Authors:** Kenji Amemiya, Yosuke Hirotsu, Yuji Iimuro, Ryosuke Tajiri, Toshio Oyama, Shuntaro Obi, Hitoshi Mochizuki, Masao Omata

**Affiliations:** ^1^ Genome Analysis Center Yamanashi Central Hospital Kofu, Yamanashi Japan; ^2^ Division of Genetics and Clinical Laboratory Yamanashi Central Hospital Kofu, Yamanashi Japan; ^3^ Department of Surgery Yamanashi Central Hospital Kofu, Yamanashi Japan; ^4^ Department of Pathology Yamanashi Central Hospital Kofu, Yamanashi Japan; ^5^ Department of Gastroenterology Yamanashi Central Hospital Kofu, Yamanashi Japan; ^6^ Department of Internal Medicine Teikyo University Chiba Medical Center Ichihara, Chiba Japan; ^7^ The University of Tokyo Tokyo Japan

**Keywords:** genomic diversity, multifocal HCCs, NGS, precision medicine

## Abstract

**Background:**

Hepatocellular carcinoma (HCC) is a primary liver cancer often associated with chronic liver disease and characterized by multifocal tumor lesions with synchronous and metachronous lesions, which poses treatment challenges due to potential genomic heterogeneity. This study aims to assess the consistency of actionable mutation profiles across synchronous and metachronous lesions in HCC patients.

**Methods:**

This study analyzed 68 patients with multifocal HCC, including 193 tumor lesions (82 synchronous, 111 metachronous). Genomic profiling of 72 HCC‐related genes was performed using next‐generation sequencing. We collected clinical and pathological data, including tumor size, grade, fibrosis, and etiology. Patients were categorized into two groups based on the consistency of actionable mutations among multifocal HCC. Statistical analyses compared clinicopathological features between these groups.

**Results:**

A total of 252 and 445 somatic mutations were identified in synchronous and metachronous tumors, respectively. Synchronous tumors had an average of 3.1 somatic mutations and 0.7 actionable mutations per lesion. Metachronous tumors had 4.0 somatic mutations and 1.0 actionable mutations per lesion. Actionable variants were found in 12 (36.4%) of 33 patients and 20 (24.4%) of 82 nodules in the synchronous tumors, and 23 (65.7%) of 35 patients and 42 (37.8%) of 111 nodules in the metachronous tumors. Compared to synchronous tumors, metachronous tumors exhibited significantly aberrant signaling pathways including the Wnt/β‐catenin (*p* = 0.009) and KEAP1/NRF2 (*p* = 0.022) pathways. There was no correlation with significant clinical differences in tumor characteristics between the consistent and divergent actionable mutation groups. Notably, divergent actionable mutations were identified in 45.6% of patients, which may be beneficial for changing potential therapies for individual tumors.

**Conclusion:**

The study shows substantial inter‐tumoral heterogeneity in multifocal HCC, indicating the necessity for comprehensive molecular profiling for tailored treatment strategies. Divergent actionable mutations across lesions suggest that a uniform treatment approach may not be effective in some patients with multifocal HCC.

## 1 Introduction

Hepatocellular carcinoma (HCC) is a primary liver cancer and a leading cause of cancer‐related mortality worldwide [1]. HCC often develops in the setting of chronic liver disease, such as viral hepatitis or cirrhosis [2, 3, 4]. A unique characteristic of HCC is the frequent occurrence of multifocal tumor lesions in the same patient [5]. These lesions may arise synchronously or metachronously [6]. Synchronous HCC, characterized by the simultaneous presence of two or more primary liver cancers at the same time, accounts for 25%–35% of all HCC patients [7, 8]. The recurrence rate after surgical resection is also high, with 30%–47% of patients recurring within 1 year, 62%–66% within 3 years, and 75%–79% within 5 years [9, 10]. These features indicate HCC patients have a potential risk of metachronous cancer.

Despite the prevalence of multifocal HCC, comprehensive molecular characterization of distinct tumor lesions within individual patients has been limited [11, 12, 13, 14, 15]. This is due to the high cost of molecular analysis, the difficulty in recruiting a sufficient number of patients, and the challenges of genomic analysis of multiple lesions. Understanding inter‐tumoral molecular diversity is essential for optimizing treatment strategies. Thus, it is important to identify actionable mutations in each nodule for precision medicine, ensuring that patients receive the most effective therapies according to their tumor profiles [16].

In Japan, the targeted therapy drug sorafenib was approved in 2009 as the first‐line treatment for advanced HCC [17]. In recent years, lenvatinib has been approved as the first‐line therapy, whereas regorafenib, ramucirumab, and cabozantinib have been approved as the second‐line treatment after sorafenib [18, 19, 20, 21, 22]. On the other hand, immune checkpoint inhibitors (ICI) have been used in the first‐line setting with the approval of atezolizumab plus bevacizumab [23]. Subsequently, durvalumab monotherapy and the durvalumab and tremelimumab combination therapy have also been approved as the first‐line treatment [24]. However, the efficacy of these therapies is limited and there is an unmet need for more effective treatments for HCC patients.

The objective of this study is to determine whether the profiles of actionable mutations are consistent or diverge in multifocal HCC. To this end, we performed comprehensive genomic profiling of multiple tumor lesions and examined the clinicopathological features between cases with consistent versus divergent actionable mutation profiles. The findings provide insights into the extent of inter‐tumoral heterogeneity in actionable mutations and its potential impact on therapeutic strategies for multifocal HCC.

## 2 Methods

### 2.1 Patients

This analysis was conducted to identify 281 patients with a pathologically confirmed HCC diagnosed at our hospital between 2009 and 2023. Among these patients, 87 patients had multiple HCC, and 68 patients provided informed consent for genomic analysis. These patients had a total of 193 tumor lesions available, comprising 82 tumor lesions from synchronous cases (*n* = 33) and 111 tumor lesions from metachronous cases (*n* = 35). Synchronous tumors were defined as multifocal tumors found at the same time, whereas metachronous tumors were defined as multifocal tumors identified at different time points [6].

### 2.2 Clinical and Pathological Data

Clinical and pathological data were extracted from medical records, including age at initial diagnosis, sex, number of tumors, maximum tumor size, main etiology, Edmondson histological grade, and degree of fibrosis. Edmondson grading and fibrosis staging were evaluated by experienced pathologists according to the established criteria [25, 26]. Intrahepatic metastasis (IM) and multicentric origin (MO) were assessed by experienced pathologists according to the established criteria in the general rules for the clinical and pathological study of primary liver cancer [27].

### 2.3 Etiology Assessment

Main etiology is classified based on virus infection status. Five milliliters of peripheral blood samples were collected in the Insepack II serum collection tubes (Sekisui Medical) and centrifuged at 3000 rpm for 5 min at room temperature. The serum was transferred to sterilized test tubes, and serum markers were measured based on the chemiluminescent immunoassay. Hepatitis B virus (HBV) infection status was determined by measuring hepatitis B surface antigen (HBsAg) levels with the Alinity i HBsAg Reagent Kit on the Alinity i system (Abbott Laboratories), with values ≥ 0.05 IU/mL considered positive. Hepatitis C virus (HCV) infection was assessed by detecting anti‐HCV antibodies with the Alinity i Anti‐HCV Reagent Kit on the Alinity i system, with a cutoff index ≥ 1.0 considered positive. Patients negative for both HBsAg and anti‐HCV were classified as non‐B non‐C.

### 2.4 Sample Processing and DNA Extraction

Peripheral blood samples were collected in EDTA‐2Na tubes and centrifuged at 820 *g* for 10 min at room temperature. Buffy coats were stored at −80°C until DNA extraction. Buffy coat DNA was extracted with the QIAamp DNA Blood Mini QIAcube Kit (Qiagen, Hilden, Germany) and the DNA concentration was determined using a NanoDrop 2000 (Thermo Fisher Scientific, Waltham, MA, USA).

Hepatocellular carcinoma tissues were fixed using 10% buffered formalin. Serial 10‐μm‐thick sections were prepared from formalin‐fixed paraffin‐embedded tissues (FFPE). Sections were stained with hematoxylin–eosin and reviewed by a pathologist to check the tumor location. To enrich the tumor content, laser capture microdissection was performed as described previously. Tumor DNA was manually extracted using the GeneRead DNA FFPE Kit (Qiagen) or automatically extracted using the MagMAX FFPE DNA/RNA Ultra Kit in the KingFisher Duo Prime (Thermo Fisher Scientific).

### 2.5 Targeted Sequencing

We searched literature and selected 72 genes related to HCC (Table S1) as described previously [28, 29, 30, 31, 32]. A total of 2842 primer pairs were designed by Ion AmpliSeq Designer contained within this panel (covering 285.5 kb). Briefly, multiplex PCR was performed using the Ion AmpliSeq Library Kit v2.0 or Ion AmpliSeq Library Kit Plus (Thermo Fisher Scientific) [33]. Primers were digested with FuPa reagent and then barcoded using Ion Xpress Barcode Adapters. Purification was performed by Agencourt AMPure XP reagents (Beckman Coulter, Brea, CA, USA) using the KingFisher Duo Prime System (Thermo Fisher Scientific). The library concentration was determined using an Ion Library Quantitation Kit. Emulsion PCR and chip loading were performed on the Ion Chef with the Ion PI Hi‐Q Chef Kit. Sequencing was performed using the Ion PI Hi‐Q Sequencing Kit on the Ion Proton Sequencer (Thermo Fisher Scientific). Targeted sequencing was also conducted on the Ion Torrent Genexus System in accordance with the manufacturer's instructions (Thermo Fisher Scientific). Amplification of DNA was performed using the aforementioned HCC panel. The Ion Torrent Genexus Library Strips and Templating Strips were incubated at room temperature for 30 min before being loaded into the sequencer. Library preparation and sequencing were conducted on the Ion Torrent Genexus System [34].

### 2.6 Data Analysis

Raw signal data from the sequencing analysis were processed using the standard pipeline in the Torrent Suite Software running on the Torrent Server or in the Genexus Software in the Ion Torrent Genexus System. The data processing pipeline involved signaling processing, base calling, quality score assignment, read alignment, quality control of mapping, and coverage analysis. Following data analysis, the annotation of somatic variants was performed by the Ion Reporter Server System (Thermo Fisher Scientific). To perform tumor–normal pair analysis, we used buffy coat DNA as a normal control for subtraction of germline mutations and to detect somatic variants in tumors. To ensure sequencing accuracy, we used the following filtering parameters for variant calling: (i) minimum number of variant allele reads ≥ 10; (ii) coverage depth ≥ 50; (iii) variant allele fraction (VAF) ≥ 0.03; (iv) UCSC Common SNPs = Not In; and (v) Confident Somatic Variants = In. The data that fell below the specified thresholds was omitted from further analysis (Table S2).

### 2.7 Actionable Mutations, Therapies and Signaling Pathway

Oncogenic variants were defined as those classified as “likely oncogenic” or “oncogenic” in the OncoKB database (last updated 01/30/2025) [35]. Actionable mutations were defined as related to corresponding potential matched drugs, which were searched for each mutation and identified in the “therapeutic” tab on OncoKB. Potential matched drugs were identified regardless of cancer type if a drug was present. Additionally, *CTNNB1* mutations were considered predictive of resistance to ICI based on previous reports [36]. The signaling pathways were classified into the Wnt/β‐catenin (*CTNNB1*, *APC*, and *AXIN1*), chromatin remodeling (*ARID1A*, *ARID1B*, and *ARID2*), PI3K/AKT/mTOR (*PIK3CA*, *PTEN*, *TSC1*, and *TSC2*), cell cycle (*TP53*, *ATM*, and *CDKN2A*), and RAS/MAPK (*KRAS* and *NF1*) [26]. It was determined that mutations classified as “likely oncogenic” or “oncogenic” in OncoKB were considered as an aberrant pathway.

### 2.8 Ethics Statement

Informed consent was obtained from all patients. This study was approved by the Institutional Review Board of clinical research and genome research committee at Yamanashi Central Hospital (G‐2018‐1) and complied with the Declaration of Helsinki principles.

### 2.9 Data Processing, Visualization, and Statistical Analysis

Interquartile range (IQR) calculations, statistical analyses, and correlation analyses were performed using R version 4.1.1 (http://www.r‐project.org/). Statistical significance was defined as a *p* value < 0.05. The following R packages were used for data cleaning and analysis: tidyverse (v2.0.0), dplyr (v1.13), stringr (1.5.0), patchwork (v1.1.1), and gtsummary (v1.5.2).

## 3 Results

### 3.1 Patient Characteristics

The study cohort consisted of 68 patients with HCC (Table S3). The median age was 73 years (interquartile range [IQR]: 66–77 years), and 51 (75%) patients were male (Table 1). Regarding viral etiology, 6 (8.8%) patients had HBV infection, 33 (49%) had HCV infection, and 29 (43%) were non‐B non‐C. Thirty‐five (51%) patients presented with metachronous tumors, whereas 33 (49%) had synchronous tumors. The median time to recurrence for metachronous tumors was 21 months (IQR: 12–37 months). The median maximum tumor size was 34 mm (IQR: 22–51 mm). Most tumors are Edmondson Grade II (*n* = 32, 47%) or Grade II–III (*n* = 25, 37%) and only Grade I–II (*n* = 2, 2.9%) or Grade III (*n* = 9, 13%). The majority of tumors exhibited fibrosis, with F4 (*n* = 21, 31%), F3 (*n* = 15, 22%), F2 (*n* = 14, 21%), F1 (*n* = 10, 15%), and F0 (*n* = 4, 11%). The clinicopathological origin was identified as IM (*n* = 29, 43%), MO (*n* = 25, 37%), and IM and MO (*n* = 14, 20%).

**TABLE 1:** Characteristics of the patients.

Characteristic	*n* = 68
*Number of tumors, n (%)*	
2	36 (53)
3	17 (25)
4	10 (15)
5	2 (2.9)
6	1 (1.5)
7	2 (2.9)
Age, median (IQR)	73 (66, 77)
*Gender, n (%)*	
F	17 (25)
M	51 (75)
*Main etiology, n (%)*	
HBV	6 (8.8)
HCV	33 (49)
NBNC	29 (43)
*Tumor timing, n (%)*	
Metachronous	35 (51)
Synchronous	33 (49)
Average period of recurrence (month),^a^ median (IQR)	21 (12, 37)
Max tumor size (mm), median (IQR)	34 (22, 51)
*Edmondson, n (%)*	
I–II	2 (2.9)
II	32 (47)
II–III	25 (37)
III	9 (13)
*Fibrosis, n (%)*	
F0	4 (5.9)
F1	10 (14.7)
F2	14 (20.6)
F3	15 (22)
F4	21 (30.9)
Unknown	4 (5.9)
*Clinicopathological origin, n (%)*	
IM	29 (43)
MO	25 (37)
IM and MO	14 (20)

Abbreviations: F, female; HBV, hepatitis B virus; HCV, hepatitis C virus; IM, intrahepatic metastasis; IQR, interquartile range; M, male; MO, multicentric origin; NBNC, non‐HBV and non‐HCV.

^a^ Average period of recurrence (months) includes data only from metachronous cases.

### 3.2 Mutational Landscape of Synchronous and Metachronous Tumors

A hallmark of HCC is the occurrence of multiple tumors, which can arise either synchronously or metachronously. To characterize the mutational landscape of these tumors, we performed next‐generation sequencing using an in‐house panel covering 72 HCC‐related genes. A total of 193 lesions were examined, including 82 lesions from synchronous and 111 lesions from metachronous HCC.

In synchronous tumors, a total of 252 somatic mutations were identified and 54 were oncogenic variants (average 3.1 somatic mutations and 0.7 actionable mutations per tumor lesion). The frequently mutated gene was *TP53* (23/82; 28%), followed by *CTNNB1* (11/82; 13%), *ARID1A* (8/82; 10%), and *ARID2* (4/82; 5%) (Table S4). Aberrant signaling pathways included cell cycle (25/82; 31%), chromatin remodeling (14/82; 17%), Wnt/β‐catenin (11/82; 13%), and PI3K/AKT/mTOR (3/82; 4%) (Table 2). In metachronous tumors, 445 somatic mutations were identified and 114 were oncogenic variants (average 4.0 somatic mutations and 1.0 actionable mutations per tumor lesion). The frequently mutated genes were *CTNNB1* (29/111; 26%), followed by *TP53* (28/111; 25%), *ARID1A* (11/111; 10%), *ARID2* (10/111; 9%), and *KEAP1* (8/111; 7%) (Table S4). Aberrant signaling pathways were predominated by cell cycle (34/111; 31%) and Wnt/β‐catenin (34/111; 31%), followed by chromatin remodeling (21/111; 19%), KEAP1/NRF2 (8/111; 7%), and PI3K/AKT/mTOR (5/111; 5%). Overall, the Wnt/β‐catenin (*p* = 0.009, Fisher's exact test) and KEAP1/NRF2 pathways (*p* = 0.022, Fisher's exact test) were found to be significantly aberrant in metachronous HCC compared to synchronous HCC (Table 2).

**TABLE 2:** Frequency of dysregulated signaling pathways in synchronous and metachronous multifocal HCC.

Signaling pathway	Synchronous, *n* (%)	Metachronous, *n* (%)	*p*
Cell cycle	25 (31)	34 (31)	1.000
Chromatin remodeling	14 (17)	21 (19)	0.889
Wnt/β‐catenin	11 (13)	34 (31)	0.009
PI3K/AKT/mTOR	3 (4)	5 (5)	1.000
Transcription	1 (1)	1 (1)	1.000
KEAP1/NRF2	0 (0)	8 (7)	0.022
RAS/MAPK	0 (0)	4 (4)	0.138

*Note:* Statistical analyses were conducted as Fisher's exact test.

### 3.3 Analysis of Clinicopathological Features Based on Actionable Mutations

**FIGURE 1:**
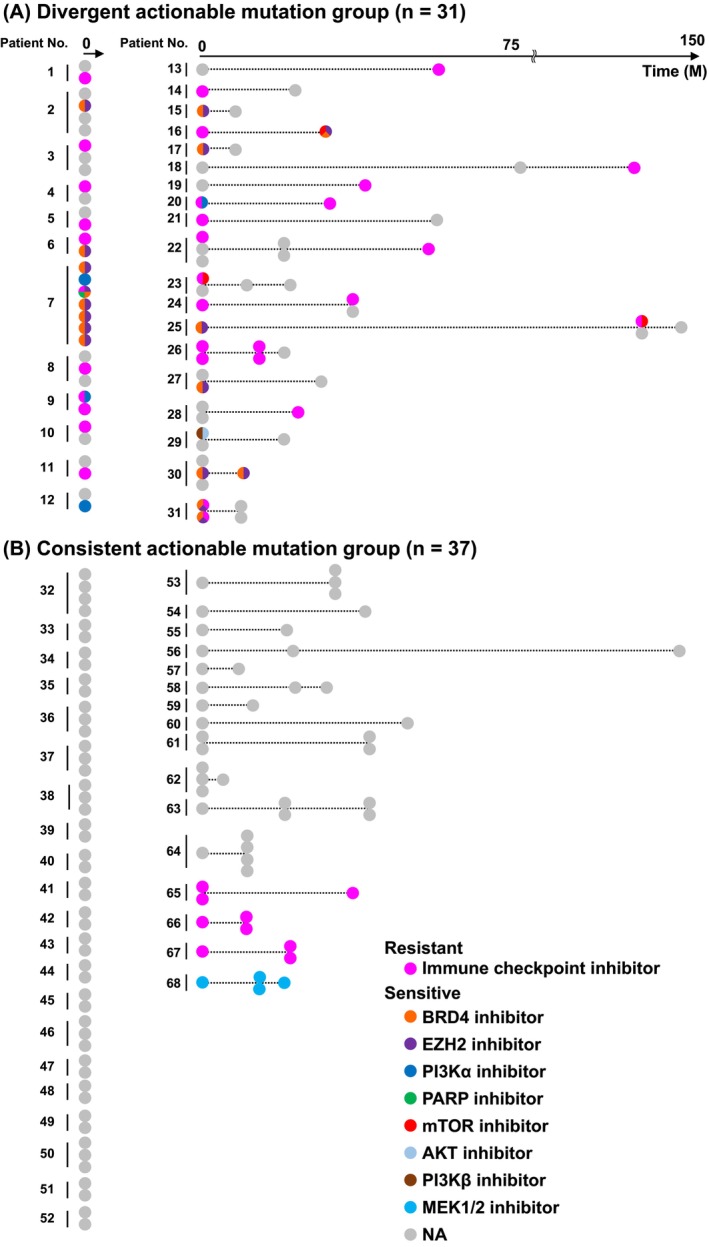
Divergent and consistent actionable mutations in multifocal HCC. The presence or absence of actionable mutations in multifocal HCC, highlighting the genomic heterogeneity and its implications for potential therapeutic options. (A) Divergent actionable mutation group (*n* = 31) and (B) consistent actionable mutation group (*n* = 37). Each circle represents a single tumor lesion. The horizontal axis indicates the timeline of tumor occurrence (M, months). Tumors with actionable mutations and corresponding potential drugs are colored: Resistance to immune checkpoint inhibitor (magenta) and sensitive to BRD4 inhibitor (orange), EZH2 inhibitor (purple), PI3Kα inhibitor (blue), PARP inhibitor (green), mTOR inhibitor (red), AKT inhibitor (light blue), PI3Kβ inhibitor (brown), and MEK1/2 inhibitor (light blue). Tumors without any actionable mutations are shown in gray. If a single tumor has multiple potential drugs, the circle is divided into multiple colors.

Identifying the presence and types of actionable mutations in HCC can guide treatment selection. We classified patients into two groups to evaluate the potential for differential treatment strategies across multiple tumors. The first group was the divergent group (*n* = 31) including patients with actionable mutations that varied between lesions. The second group was the consistent group (*n* = 37), with the same actionable mutations remaining across lesions or not detected (Figure 1 and Table S5).

Among patients in the divergent group, 39% (12/31) were synchronous cases, whereas 61% (19/31) were metachronous cases (Figure 1). In contrast, in the consistent group, 57% (21/37) were synchronous cases and 43% (16/37) were metachronous cases. In the consistent group, the majority of patients (89%; 33/37) lacked targetable mutations. In contrast, among the divergent group, most patients (74%; 23/31) had at least one tumor lesion exhibiting resistance to ICI due to alterations in the Wnt/β‐catenin pathway.

To investigate the potential clinical implications of divergent actionable mutation profiles between multifocal HCC, we performed univariable analyses comparing clinicopathological features between the divergent group and the consistent group. The median age was comparable between the consistent group (73 years, IQR: 68–77) and the divergent group (72 years, IQR: 65–76) (*p* = 0.4, Wilcoxon rank sum test) (Table 3). There were no significant differences in the number of tumors between these two groups (*p* > 0.9, Fisher's exact test). Although a higher proportion of males was observed in the divergent group (84% vs. 68%), this difference did not reach statistical significance (*p* = 0.12, Pearson's chi‐squared test). The distribution of the main etiologies was also similar between the groups (*p* = 0.6, Fisher's exact test).

**TABLE 3:** Changes in actionable mutations and clinicopathological characteristics in multiple hepatocellular carcinomas.

Characteristic	Divergent actionable mutation group (*n* = 31)	Consistent actionable mutation group (*n* = 37)	*p*
*Number of tumors, n (%)*			
2	16 (52%)	20 (54%)	> 0.9^a^
3	7 (23%)	10 (27%)	
4	5 (16%)	5 (14%)	
5	1 (3.2%)	1 (2.7%)	
6	1 (3.2%)	0 (0%)	
7	1 (3.2%)	1 (2.7%)	
Age, median (IQR)	72 (65, 76)	73 (68, 77)	0.4^b^
Gender, n (%)			
F	5 (16%)	12 (32%)	0.12^c^
M	26 (84%)	25 (68%)	
*Main etiology, n (%)*			
HBV	4 (13%)	2 (5.4%)	0.6^a^
HCV	14 (45%)	19 (51%)	
NBNC	13 (42%)	16 (43%)	
*Tumor timing, n (%)*			
Metachronous	19 (61%)	16 (43%)	0.14^c^
Synchronous	12 (39%)	21 (57%)	
Average period of recurrence (month),^a^ median (IQR)	10 (0, 25)	0 (0, 13)	0.11^b^
Max tumor size (mm), median (IQR)	35 (22, 49)	32 (22, 50)	> 0.9^b^
*Edmondson, n (%)*			
I–II	0 (0%)	2 (5.4%)	0.2^a^
II	15 (48%)	17 (46%)	
II–III	14 (45%)	11 (30%)	
III	2 (6.5%)	7 (19%)	
*Fibrosis, n (%)*			
F0	1 (3.2%)	3 (8.1%)	0.3^a^
F1	5 (16%)	5 (14%)	
F2	5 (16%)	9 (24%)	
F3	9 (29%)	6 (16%)	
F4	11 (35%)	10 (27%)	
Unknown	0 (0%)	4 (11%)	
*Clinicopathological origin, n (%)*			
IM	11 (3.2%)	18 (8.1%)	0.5^a^
MO	13 (16%)	12 (14%)	
IM and MO	7 (16%)	7 (24%)	

*Note:* Statistical analyses were conducted as follows: ^a^Fisher's exact test; ^b^Wilcoxon rank sum test; ^c^Pearson's chi‐squared test.

Abbreviations: F, female; HBV, hepatitis B virus; HCV, hepatitis C virus; IM, intrahepatic metastasis; IQR, interquartile range; M, male; MO, multicentric origin; NBNC, non‐HBV and non‐HCV.

Regarding tumor characteristics, the median time to recurrence was 0 months (IQR: 0–13) in the consistent group and 10 months (IQR: 0–25) in the divergent group, although this difference was not statistically significant (*p* = 0.11, Wilcoxon rank sum test) (Table 3). The maximum tumor size was comparable, with a median of 32 mm (IQR: 22–50 mm) in the consistent group and 35 mm (IQR: 22–49 mm) in the divergent group (*p* > 0.9, Wilcoxon rank sum test) (Table 3). There was no significant difference in the distribution of Edmondson grades between the groups (*p* = 0.2, Fisher's exact test) and the degree of fibrosis (*p* = 0.3, Fisher's exact test) (Table 3). The clinicopathological origin (IM and MO) did not show a significant difference between the two groups (*p* = 0.5, Fisher's exact test) (Table 3). These results showed that the presence of divergent actionable mutations across tumor lesions was not associated with clinically relevant differences in tumor characteristics, including time to recurrence, tumor size, differentiation, or fibrosis status. Overall, divergent actionable mutations were present in 45.6% (31/68) of patients with multifocal HCC. Collectively, the presence of divergent actionable mutations does not correspond to observable clinical differences in tumor characteristics, reinforcing the need for molecular profiling as a primary way for treatment selection.

## 4 Discussion

This study provides a comprehensive analysis of the mutational landscape and clinical implications of multifocal HCC. By examining a total of 193 tumor lesions from 68 patients, our findings revealed distinct mutational profiles between synchronous and metachronous tumors, emphasizing the need for individualized treatment approaches. Our analysis revealed that Wnt/β‐catenin and KEAP1/NRF2 aberrant pathways were observed in metachronous HCC rather than in synchronous HCC. Previous studies suggested that alterations in these pathways contribute to resistance against ICI and tyrosine kinase inhibitors, which are key therapeutic options for advanced HCC. Wnt/β‐catenin activation promotes immune evasion by suppressing the infiltration of CD8‐positive cytotoxic T cells and antigen‐presenting dendritic cells into the tumor microenvironment, leading to an immune‐excluded phenotype [36, 37, 38]. KEAP1/NRF2 dysregulation enhances antioxidant defenses and enables tumor cells to resist reactive oxygen species‐induced apoptosis [39, 40, 41, 42]. This leads to a decrease in the efficacy of tyrosine kinase inhibitor treatment [43, 44]. These findings suggest that metachronous HCC may be more resistant to standard therapies because it has a higher frequency of Wnt/β‐catenin and KEAP1/NRF2 dysfunctions.

Our previous studies in lung and gastric cancers show the importance of analyzing the genomic landscape across distinct lesions within a patient with multiple tumors [45, 46, 47]. This approach is currently limited in HCC [48]; therefore, we analyzed multifocal HCC. We found that about half of the patients (45.6%) harbored divergent actionable mutations across their tumor lesions [16]. This finding indicates the importance of comprehensive genomic profiling for each lesion to optimize treatment selection based on the molecular profile in tumor. The presence of divergent actionable mutations was not associated with clinically relevant differences in several characteristics, including time to recurrence, main etiology, tumor size, differentiation, or fibrosis status. This finding suggests that genomic heterogeneity may not directly correlate with clinicopathological features [13] (Table 3). Although this study did not directly control for etiology due to the limited sample size, no significant differences in HBV/HCV status were observed between the groups. Future studies should be needed to investigate the impact of these variables on genomic heterogeneity in HCC. The presence of a substantial proportion of cases with divergent actionable mutations across lesions suggests that a one‐size‐fits‐all treatment approach may not be optimal for patients with multifocal HCC.

Tailoring therapeutic strategies to target the specific actionable mutations in each lesion could potentially improve clinical outcomes. In the divergent group, distinct therapeutic approaches may be required for each tumor lesion, increasing the complexity of treatment planning. The divergent group pointed out that selecting treatments and managing adverse reactions could be difficult. Conversely, in the consistent group, a targeted monotherapy may be a more reasonable option. These findings suggest that understanding tumor‐specific actionable mutations could provide clinically valuable insights, aiding in optimizing therapeutic strategies for multifocal HCC.

To date, many targeted therapies for specific genetic mutations are not yet approved for HCC, indicating there are further needs for drug development to provide effective treatment options [49, 50, 51]. However, with the future approval of more targeted therapies for HCC, the benefits of adapting treatment strategies based on comprehensive genomic profiling will become increasingly significant [52, 53, 54]. To this end, multiple biopsies should be obtained to analyze the genomic profile of each tumor. In addition, based on the identified actionable or resistance mutations, clinicians can offer tailored treatment for each patient and reassess to optimize treatments during the course of therapies.

Our study has several limitations. First, the sample size is relatively small. To validate our findings, larger studies are needed. Second, this study was conducted in a single institution, which likely minimized variability in pre‐analytical processing. However, future multicenter studies should examine how differences in sample handling may affect the results. Third, we were unable to examine patient outcomes when treatment options were changed based on the actionable mutations. Further investigation is needed to elucidate the functional implications of specific mutations and their effect on treatment outcomes.

In summary, this study demonstrates significant inter‐tumoral genomic heterogeneity in multifocal HCC. These findings illustrate the clinical implications of comprehensive molecular profiling for guiding personalized treatment and improving outcomes. Although the clinical trials are necessary, our results support the integration of molecular diagnostics into clinical practice to improve treatment strategies for patients with multifocal HCC, ultimately advancing the field of tumor‐level precision oncology.

## Author Contributions

K.A. data curation, formal analysis, investigation, visualization, writing – original draft, writing – review and editing, and funding acquisition. Y.H. conceptualization, data curation, formal analysis, resources, writing – original draft, writing – review and editing, visualization, and funding acquisition. Y.I. investigation, resources. R.T. investigation, resources. T.O. investigation, resources. S.O. investigation, resources. H.M. investigation, resources, project administration. M.O. conceptualization, supervision, and writing – review and editing. All authors reviewed the manuscript.

## Conflicts of Interest

The authors declare no conflicts of interest.

## Supporting information


Table S1.

Table S2.

Table S3.

Table S4.

Table S5.

## Data Availability

The source data underlying figures and tables are available upon reasonable request from the corresponding author.
