# Supplementary material for: Decoding Genomic Diversity to Guide Tumor Lesion‐Specific Treatment of Multifocal Hepatocellular Carcinoma
Source: Cancer Med. 2025 Mar 27;14(7):e70814. doi: 10.1002/cam4.70814 (PMC11947740; doi:10.1002/cam4.70814)
Supplement: Supplementary file 2 — Table S2. [file CAM4-14-e70814-s003.docx]

**Supplementary Table 2. Patients information in this study**

| **Patient number** | **Number of tumors** | **Age** | **Gender** | **Main etiology** | **Tumor timing** | **Average period of recurrence (month)** | **Max tumor size (mm)** | **Edmondson** | **Fibrosis** | **Actionable mutations varied among tumors** |
| --- | --- | --- | --- | --- | --- | --- | --- | --- | --- | --- |
| 1 | 2 | 53 | M | HCV | S | 0 | 15 | II-III | F4 | Yes |
| 2 | 4 | 78 | M | NBNC | S | 0 | 20 | II | F4 | Yes |
| 3 | 3 | 79 | M | NBNC | S | 0 | 40 | II-III | F2 | Yes |
| 4 | 2 | 72 | M | NBNC | S | 0 | 25 | II | F3 | Yes |
| 5 | 2 | 58 | M | NBNC | S | 0 | 35 | II-III | F2 | Yes |
| 6 | 2 | 63 | M | HCV | S | 0 | 20 | II-III | F3 | Yes |
| 7 | 7 | 76 | M | NBNC | S | 0 | 120 | II-III | F3 | Yes |
| 8 | 3 | 73 | M | HCV | S | 0 | 22 | II | F1 | Yes |
| 9 | 2 | 71 | M | NBNC | S | 0 | 90 | II | F1 | Yes |
| 10 | 2 | 78 | M | NBNC | S | 0 | 42 | II | F3 | Yes |
| 11 | 2 | 74 | M | HCV | S | 0 | 32 | II-III | F1 | Yes |
| 12 | 2 | 66 | M | HCV | S | 0 | 19 | III | F4 | Yes |
| 13 | 2 | 73 | M | HCV | M | 59 | 20 | II | F0 | Yes |
| 14 | 2 | 76 | M | HBV | M | 21 | 35 | II | F2 | Yes |
| 15 | 2 | 74 | F | HCV | M | 9 | 95 | III | F4 | Yes |
| 16 | 2 | 71 | F | HBV | M | 26 | 35 | II-III | F4 | Yes |
| 17 | 2 | 70 | M | HBV | M | 9 | 20 | II | F4 | Yes |
| 18 | 3 | 76 | M | HCV | M | 37 | 28 | II | F4 | Yes |
| 19 | 2 | 65 | M | NBNC | M | 59 | 20 | II-III | F4 | Yes |
| 20 | 2 | 76 | M | NBNC | M | 16 | 60 | II-III | F1 | Yes |
| 21 | 2 | 59 | F | HBV | M | 56 | 40 | II-III | F3 | Yes |
| 22 | 6 | 72 | M | NBNC | M | 28 | 70 | II | F3 | Yes |
| 23 | 4 | 83 | M | HCV | M | 12 | 55 | II-III | F4 | Yes |
| 24 | 3 | 85 | M | NBNC | M | 37 | 65 | II | F1 | Yes |
| 25 | 4 | 62 | M | HCV | M | 73 | 60 | II-III | F2 | Yes |
| 26 | 5 | 65 | M | NBNC | M | 10 | 26 | II-III | F3 | Yes |
| 27 | 3 | 61 | M | HCV | M | 24 | 22 | II-III | F4 | Yes |
| 28 | 3 | 80 | F | HCV | M | 22 | 40 | II | F3 | Yes |
| 29 | 3 | 63 | M | NBNC | M | 16 | 30 | II | F3 | Yes |
| 30 | 4 | 66 | M | HCV | M | 10 | 37 | II | F4 | Yes |
| 31 | 4 | 77 | F | HCV | M | 10 | 30 | II | F2 | Yes |
| 32 | 4 | 76 | M | HBV | S | 0 | 38 | III | F2 | No |
| 33 | 2 | 77 | M | HCV | S | 0 | 22 | III | F3 | No |
| 34 | 2 | 54 | M | NBNC | S | 0 | 12 | II | F4 | No |
| 35 | 2 | 79 | M | HCV | S | 0 | 50 | III | F2 | No |
| 36 | 3 | 73 | F | NBNC | S | 0 | 40 | II | F2 | No |
| 37 | 3 | 68 | M | HCV | S | 0 | 32 | II-III | F4 | No |
| 38 | 3 | 82 | M | HCV | S | 0 | 70 | III | F4 | No |
| 39 | 2 | 71 | M | HCV | S | 0 | 17 | I-II | Unkown | No |
| 40 | 2 | 68 | F | HCV | S | 0 | 19 | II-III | F3 | No |
| 41 | 2 | 82 | M | NBNC | S | 0 | 30 | II-III | F1 | No |
| 42 | 2 | 73 | M | NBNC | S | 0 | 20 | II-III | F3 | No |
| 43 | 2 | 75 | F | NBNC | S | 0 | 15 | II-III | F4 | No |
| 44 | 2 | 71 | M | NBNC | S | 0 | 20 | I-II | F3 | No |
| 45 | 2 | 73 | M | HCV | S | 0 | 80 | II | F2 | No |
| 46 | 3 | 73 | F | NBNC | S | 0 | 45 | II-III | F4 | No |
| 47 | 2 | 65 | F | HBV | S | 0 | 25 | II | F2 | No |
| 48 | 2 | 84 | M | NBNC | S | 0 | 35 | II-III | F2 | No |
| 49 | 2 | 73 | M | HCV | S | 0 | 25 | II | F4 | No |
| 50 | 3 | 80 | M | NBNC | S | 0 | 45 | II | F2 | No |
| 51 | 2 | 70 | M | HCV | S | 0 | 22 | II | F4 | No |
| 52 | 2 | 71 | M | NBNC | S | 0 | 30 | II | F4 | No |
| 53 | 4 | 57 | F | NBNC | M | 31 | 12 | II | F2 | No |
| 54 | 2 | 74 | F | HCV | M | 42 | 30 | II-III | Unkown | No |
| 55 | 2 | 55 | M | HCV | M | 18 | 25 | III | Unkown | No |
| 56 | 3 | 72 | M | HCV | M | 48 | 90 | III | F1 | No |
| 57 | 2 | 90 | F | NBNC | M | 4 | 55 | II-III | F1 | No |
| 58 | 3 | 86 | F | HCV | M | 9 | 30 | II | F3 | No |
| 59 | 2 | 65 | M | HCV | M | 12 | 40 | II | Unkown | No |
| 60 | 2 | 72 | F | HCV | M | 49 | 20 | II | F3 | No |
| 61 | 4 | 65 | F | HCV | M | 42 | 25 | III | F4 | No |
| 62 | 4 | 76 | M | HCV | M | 3 | 90 | II-III | F4 | No |
| 63 | 7 | 77 | F | HCV | M | 15 | 55 | II | F1 | No |
| 64 | 5 | 80 | M | HCV | M | 13 | 50 | II | F1 | No |
| 65 | 3 | 65 | M | NBNC | M | 37 | 120 | II-III | F2 | No |
| 66 | 3 | 86 | M | NBNC | M | 13 | 40 | II | F0 | No |
| 67 | 3 | 60 | M | NBNC | M | 21 | 170 | II | F0 | No |
| 68 | 4 | 71 | M | NBNC | M | 11 | 60 | II | F0 | No |

M, male; F, female; HBV, hepatitis B virus; HCV, hepatitis C virus; NBNC, non-HBV and non-HCV; S, Syncrhonous; M, Metacrhonous.
