# Supplementary material for: Decoding Genomic Diversity to Guide Tumor Lesion‐Specific Treatment of Multifocal Hepatocellular Carcinoma
Source: Cancer Med. 2025 Mar 27;14(7):e70814. doi: 10.1002/cam4.70814 (PMC11947740; doi:10.1002/cam4.70814)
Supplement: Supplementary file 4 — Table S4. [file CAM4-14-e70814-s002.docx]

**Supplementary Table 4. Frequency of genes with oncogenic mutation in synchronous and metachronous multifocal HCC**

| **Genes** | **Signaling pathway** | **Synchronous, n (%)** | **Metachronous, n (%)** | **P value** |
| --- | --- | --- | --- | --- |
| *TP53* | Cell cycle | 23 (28%) | 28 (25%) | 0.784 |
| *CTNNB1* | Wnt/β-catenin | 11 (13%) | 29 (26%) | 0.048 |
| *ARID1A* | Chromatin remodeling | 8 (10%) | 11 (10%) | 1.000 |
| *ARID2* | Chromatin remodeling | 4 (5%) | 10 (9%) | 0.401 |
| *PIK3CA* | PI3K/AKT/mTOR | 3 (4%) | 2 (2%) | 0.652 |
| *CDKN1A* | Cell cycle | 1 (1%) | 4 (4%) | 0.397 |
| *KMT2B* | Chromatin remodeling | 1 (1%) | 0 (0%) | 0.425 |
| *ATM* | Cell cycle | 1 (1%) | 0 (0%) | 0.425 |
| *MEN1* | Transcription | 1 (1%) | 0 (0%) | 0.425 |
| *SMARCA4* | PI3K/AKT/mTOR | 1 (1%) | 0 (0%) | 0.425 |
| *KEAP1* | KEAP1/NRF2 | 0 (0%) | 8 (7%) | 0.022 |
| *TSC2* | PI3K/AKT/mTOR | 0 (0%) | 4 (4%) | 0.138 |
| *NF1* | RAS/MAPK | 0 (0%) | 4 (4%) | 0.138 |
| *AXIN1* | Wnt/β-catenin | 0 (0%) | 2 (2%) | 0.509 |
| *APC* | Wnt/β-catenin | 0 (0%) | 2 (2%) | 0.509 |
| *RB1* | Cell cycle | 0 (0%) | 2 (2%) | 0.509 |
| *SMARCA2* | PI3K/AKT/mTOR | 0 (0%) | 2 (2%) | 0.509 |
| *KMT2B* | Chromatin remodeling | 0 (0%) | 1 (1%) | 1.000 |
| *NCOR1* | Wnt/β-catenin | 0 (0%) | 1 (1%) | 1.000 |
| *ARID1B* | Chromatin remodeling | 0 (0%) | 1 (1%) | 1.000 |
| *RPS6KA3* | Cell cycle | 0 (0%) | 1 (1%) | 1.000 |
| *PTEN* | PI3K/AKT/mTOR | 0 (0%) | 1 (1%) | 1.000 |
| *TCF7L2* | Transcription | 0 (0%) | 1 (1%) | 1.000 |

Statistical analyses were conducted as Fisher's exact test.
