# Supplementary material for: Decoding Genomic Diversity to Guide Tumor Lesion‐Specific Treatment of Multifocal Hepatocellular Carcinoma
Source: Cancer Med. 2025 Mar 27;14(7):e70814. doi: 10.1002/cam4.70814 (PMC11947740; doi:10.1002/cam4.70814)
Supplement: Supplementary file 5 — Table S5. [file CAM4-14-e70814-s005.docx]

**Supplementary Table 5. Actionable mutations and potential matched drugs**

| **Patient No.** | **Tumor No.** | **Gene** | **Mutation** | **Potential matched drugs** | **Classification in Figure 1** | **Therapeutic Level** | **FDA Level** | **Cancer type** |
| --- | --- | --- | --- | --- | --- | --- | --- | --- |
| 1 | T2 | *CTNNB1* | p.Ser45Pro | Resistance to Atezolizumab and nivolumab | Resistance to ICI | None | None | All solid tumors |
| 2 | T2 | *ARID1A* | p.Asp1286Ter | PLX2853 | BRD4 inhibitor | 4 | 3 | All solid tumors |
|  |  |  |  | Tazemetostat | EZH2 inhibitor | 4 | 3 | All solid tumors |
| 3 | T1 | *CTNNB1* | p.Thr41Ile | Resistance to Atezolizumab and nivolumab | Resistance to ICI | None | None | All solid tumors |
| 4 | T1 | *CTNNB1* | p.Ser45Ala | Resistance to Atezolizumab and nivolumab | Resistance to ICI | None | None | All solid tumors |
| 5 | T2 | *CTNNB1* | p.Gly34Arg | Resistance to Atezolizumab and nivolumab | Resistance to ICI | None | None | All solid tumors |
| 6 | T1 | *CTNNB1* | p.Ser37Cys | Resistance to Atezolizumab and nivolumab | Resistance to ICI | None | None | All solid tumors |
|  | T2 | *ARID1A* | p.Tyr1362fs | PLX2853 | BRD4 inhibitor | 4 | 3 | All solid tumors |
|  |  |  |  | Tazemetostat | EZH2 inhibitor | 4 | 3 | All solid tumors |
| 7 | T1 | *ARID1A* | p.Leu1695fs | PLX2853 | BRD4 inhibitor | 4 | 3 | All solid tumors |
|  |  |  |  | Tazemetostat | EZH2 inhibitor | 4 | 3 | All solid tumors |
|  | T2 | *PIK3CA* | p.His1047Arg | LOXO-783 | PI3Kα inhibitor | 4 | 3 | All solid tumors |
|  |  |  |  | RLY-2608 | PI3Kα inhibitor | 4 | 3 | All solid tumors |
|  |  |  |  | RLY-2608 + Fulvestrant | PI3Kα inhibitor | 4 | 2 | Breast Cancer |
|  |  |  |  | LOXO-783 + Fulvestrant | PI3Kα inhibitor | 4 | 2 | Breast Cancer |
|  |  |  |  | LOXO-783 + LY3484356 | PI3Kα inhibitor | 4 | 2 | Breast Cancer |
|  |  |  |  | LOXO-783 + Abemaciclib + Fulvestrant, | PI3Kα inhibitor | 4 | 2 | Breast Cancer |
|  |  |  |  | LOXO-783 + Paclitaxel | PI3Kα inhibitor | 4 | 2 | Breast Cancer |
|  |  |  |  | LOXO-783 + Abemaciclib + Aromatase Inhibition Therapy | PI3Kα inhibitor | 4 | 2 | Breast Cancer |
|  |  |  |  | LOXO-783 + Abemaciclib + LY3484356 | PI3Kα inhibitor | 4 | 2 | Breast Cancer |
|  | T3 | *ARID1A* | p.Gln1399Ter | PLX2853 | BRD4 inhibitor | 4 | 3 | All solid tumors |
|  |  |  |  | Tazemetostat | EZH2 inhibitor | 4 | 3 | All solid tumors |
|  |  | *ATM* | p.Trp2638Ter | Olaparib | PARP inhibitor | 1 | 2 | Prostate Cancer |
|  |  |  |  | Talazoparib + Enzalutamide | PARP inhibitor | 1 | 2 | Prostate Cancer |
|  |  | *CTNNB1* | p.Gly34Arg | Resistance to Atezolizumab and nivolumab | Resistance to ICI | None | None | All solid tumors |
|  | T4 | *ARID1A* | p.Leu1695fs | PLX2853 | BRD4 inhibitor | 4 | 3 | All solid tumors |
|  |  |  |  | Tazemetostat | EZH2 inhibitor | 4 | 3 | All solid tumors |
|  | T5 | *ARID1A* | p.Leu1695fs | PLX2853 | BRD4 inhibitor | 4 | 3 | All solid tumors |
|  |  |  |  | Tazemetostat | EZH2 inhibitor | 4 | 3 | All solid tumors |
|  | T6 | *ARID1A* | p.Leu1695fs | PLX2853 | BRD4 inhibitor | 4 | 3 | All solid tumors |
|  |  |  |  | Tazemetostat | EZH2 inhibitor | 4 | 3 | All solid tumors |
|  | T7 | *ARID1A* | p.Leu1695fs | PLX2853 | BRD4 inhibitor | 4 | 3 | All solid tumors |
|  |  |  |  | Tazemetostat | EZH2 inhibitor | 4 | 3 | All solid tumors |
| 8 | T2 | *CTNNB1* | p.Ser45Phe | Resistance to Atezolizumab and nivolumab I | Resistance to ICI | None | None | All solid tumors |
| 9 | T1 | *CTNNB1* | p.Thr41Ile | Resistance to Atezolizumab and nivolumab | Resistance to ICI | None | None | All solid tumors |
|  |  | *PIK3CA* | p.His1047Arg | LOXO-783 | PI3Kα inhibitor | 4 | 3 | All solid tumors |
|  |  |  |  | RLY-2608 | PI3Kα inhibitor | 4 | 3 | All solid tumors |
|  |  |  |  | RLY-2608 + Fulvestrant | PI3Kα inhibitor | 4 | 2 | Breast Cancer |
|  |  |  |  | LOXO-783 + Fulvestrant | PI3Kα inhibitor | 4 | 2 | Breast Cancer |
|  |  |  |  | LOXO-783 + LY3484356 | PI3Kα inhibitor | 4 | 2 | Breast Cancer |
|  |  |  |  | LOXO-783 + Abemaciclib + Fulvestrant, | PI3Kα inhibitor | 4 | 2 | Breast Cancer |
|  |  |  |  | LOXO-783 + Paclitaxel | PI3Kα inhibitor | 4 | 2 | Breast Cancer |
|  |  |  |  | LOXO-783 + Abemaciclib + Aromatase Inhibition Therapy | PI3Kα inhibitor | 4 | 2 | Breast Cancer |
|  |  |  |  | LOXO-783 + Abemaciclib + LY3484356 | PI3Kα inhibitor | 4 | 2 | Breast Cancer |
|  | T2 | *CTNNB1* | p.Thr41Ile | Resistance to Atezolizumab and nivolumab | Resistance to ICI | None | None | All solid tumors |
| 10 | T1 | *CTNNB1* | p.Thr41Ile | Resistance to Atezolizumab and nivolumab | Resistance to ICI | None | None | All solid tumors |
| 11 | T2 | *CTNNB1* | p.Ser45Phe | Resistance to Atezolizumab and nivolumab | Resistance to ICI | None | None | All solid tumors |
| 12 | T2 | *PIK3CA* | p.Glu545Ala | Alpelisib + Fulvestrant | PI3Kα inhibitor | 1 | 2 | Breast Cancer |
|  |  |  |  | RLY-2608 | PI3Kα inhibitor | 4 | 3 | All solid tumors |
|  |  |  |  | RLY-2608 + Fulvestrant | PI3Kα inhibitor | 4 | 2 | Breast Cancer |
| 13 | T2 | *CTNNB1* | p.His36Pro | Resistance to Atezolizumab and nivolumab | Resistance to ICI | None | None | All solid tumors |
| 14 | T1 | *CTNNB1* | p.Thr41Ala | Resistance to Atezolizumab and nivolumab | Resistance to ICI | None | None | All solid tumors |
| 15 | T1 | *ARID1A* | p.His1541fs | PLX2853 | BRD4 inhibitor | 4 | 3 | All solid tumors |
|  |  |  |  | Tazemetostat | EZH2 inhibitor | 4 | 3 | All solid tumors |
| 16 | T1 | *CTNNB1* | p.Gly34Val | Resistance to Atezolizumab and nivolumab | Resistance to ICI | None | None | All solid tumors |
|  | T2 | *ARID1A* | p.Ala2235fs | PLX2853 | BRD4 inhibitor | 4 | 3 | All solid tumors |
|  |  |  |  | Tazemetostat | EZH2 inhibitor | 4 | 3 | All solid tumors |
|  |  | *TSC2* | p.Val391fs | Everolimus | mTOR inhibitor | 1 | 2 | Encapsulated Glioma |
|  |  |  |  | ABI-009 | mTOR inhibitor | 3A | 3 | Perivascular Epithelioid Cell Tumor, Uterine Perivascular Epithelioid Cell Tumor |
| 17 | T1 | *ARID1A* | p.Pro728fs | PLX2853 | BRD4 inhibitor | 4 | 3 | All solid tumors |
|  |  |  |  | Tazemetostat | EZH2 inhibitor | 4 | 3 | All solid tumors |
|  |  |  | p.Arg1446Ter | PLX2853 | BRD4 inhibitor | 4 | 3 | All solid tumors |
|  |  |  |  | Tazemetostat | EZH2 inhibitor | 4 | 3 | All solid tumors |
| 18 | T3 | *CTNNB1* | p.Thr41Ala | Resistance to Atezolizumab and nivolumab | Resistance to ICI | None | None | All solid tumors |
| 19 | T2 | *CTNNB1* | p.Ser33Cys | Resistance to Atezolizumab and nivolumab | Resistance to ICI | None | None | All solid tumors |
| 20 | T1 | *PIK3CA* | p.His1047Arg | LOXO-783 | PI3Kα inhibitor | 4 | 3 | All solid tumors |
|  |  |  |  | RLY-2608 | PI3Kα inhibitor | 4 | 3 | All solid tumors |
|  |  |  |  | RLY-2608 + Fulvestrant | PI3Kα inhibitor | 4 | 2 | Breast Cancer |
|  |  |  |  | LOXO-783 + Fulvestrant | PI3Kα inhibitor | 4 | 2 | Breast Cancer |
|  |  |  |  | LOXO-783 + LY3484356 | PI3Kα inhibitor | 4 | 2 | Breast Cancer |
|  |  |  |  | LOXO-783 + Abemaciclib + Fulvestrant, | PI3Kα inhibitor | 4 | 2 | Breast Cancer |
|  |  |  |  | LOXO-783 + Paclitaxel | PI3Kα inhibitor | 4 | 2 | Breast Cancer |
|  |  |  |  | LOXO-783 + Abemaciclib + Aromatase Inhibition Therapy | PI3Kα inhibitor | 4 | 2 | Breast Cancer |
|  |  |  |  | LOXO-783 + Abemaciclib + LY3484356 | PI3Kα inhibitor | 4 | 2 | Breast Cancer |
|  |  | *CTNNB1* | p.Ser37Cys | Resistance to Atezolizumab and nivolumab | Resistance to ICI | None | None | All solid tumors |
|  |  | *PIK3CA* | p.Glu545Lys | Alpelisib + Fulvestrant | PI3Kα inhibitor | 1 | 2 | Breast Cancer |
|  |  |  |  | RLY-2608 | PI3Kα inhibitor | 4 | 3 | All solid tumors |
|  |  |  |  | RLY-2608 + Fulvestrant | PI3Kα inhibitor | 4 | 2 | Breast Cancer |
| 21 | T1 | *CTNNB1* | p.Thr41Ala | Resistance to Atezolizumab and nivolumab | Resistance to ICI | None | None | All solid tumors |
| 22 | T1 | *CTNNB1* | p.Ser45Pro | Resistance to Atezolizumab and nivolumab | Resistance to ICI | None | None | All solid tumors |
|  | T6 | *CTNNB1* | p.Ser37Cys | Resistance to Atezolizumab and nivolumab | Resistance to ICI | None | None | All solid tumors |
| 23 | T1 | *TSC2* | p.Tyr324Ter | Everolimus | mTOR inhibitor | 1 | 2 | Encapsulated Glioma |
|  |  |  |  | ABI-009 | mTOR inhibitor | 3A | 3 | Perivascular Epithelioid Cell Tumor,  Uterine Perivascular Epithelioid Cell Tumor |
|  |  |  | p.Ser1094Ter | Everolimus | mTOR inhibitor | 1 | 2 | Encapsulated Glioma |
|  |  |  |  | ABI-009 | mTOR inhibitor | 3A | 3 | Perivascular Epithelioid Cell Tumor, Uterine Perivascular Epithelioid Cell Tumor |
|  |  | *CTNNB1* | p.Ile35Ser | Resistance to Atezolizumab and nivolumab | Resistance to ICI | None | None | All solid tumors |
| 24 | T1 | *CTNNB1* | p.Ser33Pro | Resistance to Atezolizumab and nivolumab | Resistance to ICI | None | None | All solid tumors |
|  | T2 | *CTNNB1* | p.Gly34Glu | Resistance to Atezolizumab and nivolumab | Resistance to ICI | None | None | All solid tumors |
| 25 | T1 | *ARID1A* | p.Gln1486fs | PLX2853 | BRD4 inhibitor | 4 | 3 | All solid tumors |
|  |  |  |  | Tazemetostat | EZH2 inhibitor | 4 | 3 | All solid tumors |
|  | T2 | *TSC2* | p.Asn1681fs | Everolimus | mTOR inhibitor | 1 | 2 | Encapsulated Glioma |
|  |  |  |  | ABI-009 | mTOR inhibitor | 3A | 3 | Perivascular Epithelioid Cell Tumor, Uterine Perivascular Epithelioid Cell Tumor |
|  |  | *CTNNB1* | p.Ser33Tyr | Resistance to Atezolizumab and nivolumab | Resistance to ICI | None | None | All solid tumors |
| 26 | T1 | *CTNNB1* | p.Gly34Arg | Resistance to Atezolizumab and nivolumab | Resistance to ICI | None | None | All solid tumors |
|  | T2 | *CTNNB1* | p.Gly34Arg | Resistance to Atezolizumab and nivolumab | Resistance to ICI | None | None | All solid tumors |
|  | T3 | *CTNNB1* | p.Gly34Arg | Resistance to Atezolizumab and nivolumab | Resistance to ICI | None | None | All solid tumors |
|  | T4 | *CTNNB1* | p.Gly34Arg | Resistance to Atezolizumab and nivolumab | Resistance to ICI | None | None | All solid tumors |
| 27 | T2 | *ARID1A* | p.Leu445fs | PLX2853 | BRD4 inhibitor | 4 | 3 | All solid tumors |
|  |  |  |  | Tazemetostat | EZH2 inhibitor | 4 | 3 | All solid tumors |
| 28 | T3 | *CTNNB1* | p.Ser37Pro | Resistance to Atezolizumab and nivolumab | Resistance to ICI | None | None | All solid tumors |
| 29 | T1 | *PTEN* | p.Trp274Ter | Capivasertib + Fulvestrant | AKT inhibitor | 1 | 2 | Breast Cancer |
|  |  |  |  | GSK2636771, AZD8186 | PI3Kβ inhibitor | 4 | 3 | All solid tumors |
| 30 | T2 | *ARID1A* | p.Asp1286Ter | PLX2853 | BRD4 inhibitor | 4 | 3 | All solid tumors |
|  |  |  |  | Tazemetostat | EZH2 inhibitor | 4 | 3 | All solid tumors |
|  | T4 | *ARID1A* | p.Asp1286Ter | PLX2853 | BRD4 inhibitor | 4 | 3 | All solid tumors |
|  |  |  |  | Tazemetostat | EZH2 inhibitor | 4 | 3 | All solid tumors |
| 31 | T1 | *CTNNB1* | p.Thr41Ala | Resistance to ICI | Resistance to ICI | None | None | All solid tumors |
|  |  | *ARID1A* | p.Gly243fs | PLX2853 | BRD4 inhibitor | 4 | 3 | All solid tumors |
|  |  |  |  | Tazemetostat | EZH2 inhibitor | 4 | 3 | All solid tumors |
|  | T2 | *CTNNB1* | p.Thr41Ala | Resistance to Atezolizumab and nivolumab | Resistance to ICI | None | None | All solid tumors |
|  |  | *ARID1A* | p.Gly243fs | PLX2853 | BRD4 inhibitor | 4 | 3 | All solid tumors |
|  |  |  |  | Tazemetostat | EZH2 inhibitor | 4 | 3 | All solid tumors |
|  |  | *ARID1A* | p.Glu1668fs | PLX2853 | BRD4 inhibitor | 4 | 3 | All solid tumors |
|  |  |  |  | Tazemetostat | EZH2 inhibitor | 4 | 3 | All solid tumors |
| 65 | T1 | *CTNNB1* | p.Asp32Tyr | Resistance to Atezolizumab and nivolumab | Resistance to ICI | None | None | All solid tumors |
|  | T2 | *CTNNB1* | p.Asp32Tyr | Resistance to Atezolizumab and nivolumab | Resistance to ICI | None | None | All solid tumors |
|  | T3 | *CTNNB1* | p.Asp32Tyr | Resistance to Atezolizumab and nivolumab | Resistance to ICI | None | None | All solid tumors |
| 66 | T1 | *CTNNB1* | p.Ser37Phe | Resistance to Atezolizumab and nivolumab | Resistance to ICI | None | None | All solid tumors |
|  | T2 | *CTNNB1* | p.Ser37Phe | Resistance to Atezolizumab and nivolumab | Resistance to ICI | None | None | All solid tumors |
|  | T3 | *CTNNB1* | p.Ser37Phe | Resistance to Atezolizumab and nivolumab | Resistance to ICI | None | None | All solid tumors |
| 67 | T1 | *CTNNB1* | p.Ser45Pro | Resistance to Atezolizumab and nivolumab | Resistance to ICI | None | None | All solid tumors |
|  | T2 | *CTNNB1* | p.Ser45Pro | Resistance to Atezolizumab and nivolumab | Resistance to ICI | None | None | All solid tumors |
|  | T3 | *CTNNB1* | p.Ser45Pro | Resistance to Atezolizumab and nivolumab | Resistance to ICI | None | None | All solid tumors |
| 68 | T1 | *NF1* | p.Trp696Ter | Selumetinib | MEK1/2 inhibitor | 1 | 2 | Neurofibroma |
|  |  |  |  | Cobimetinib, Trametinib | MEK1/2 inhibitor | 4 | 3 | All solid tumors |
|  | T2 | *NF1* | p.Trp696Ter | Selumetinib | MEK1/2 inhibitor | 1 | 2 | Neurofibroma |
|  |  |  |  | Cobimetinib, Trametinib | MEK1/2 inhibitor | 4 | 3 | All solid tumors |
|  | T3 | *NF1* | p.Trp696Ter | Selumetinib | MEK1/2 inhibitor | 1 | 2 | Neurofibroma |
|  |  |  |  | Cobimetinib, Trametinib | MEK1/2 inhibitor | 4 | 3 | All solid tumors |
|  | T4 | *NF1* | p.Trp696Ter | Selumetinib | MEK1/2 inhibitor | 1 | 2 | Neurofibroma |
|  |  |  |  | Cobimetinib, Trametinib | MEK1/2 inhibitor | 4 | 3 | All solid tumors |

ICI; immune checkpoint inhibitor
